# The European Perspective on the Management of Acute Major Hemorrhage and Coagulopathy after Trauma: Summary of the 2019 Updated European Guideline

**DOI:** 10.3390/jcm10020362

**Published:** 2021-01-19

**Authors:** Marc Maegele

**Affiliations:** Department of Traumatology and Orthopedic Surgery, Cologne-Merheim Medical Center (CMMC), Institute for Research in Operative Medicine (IFOM), University Witten-Herdecke, D-51109 Cologne, Germany; Marc.Maegele@t-online.de; Tel.: +49-(0)-221-89-07-13-614; Fax: +49-(0)-221-89-07-30-85

**Keywords:** trauma, hemorrhage, coagulopathgy, guideline

## Abstract

Non-controlled hemorrhage with accompanying trauma-induced coagulopathy (TIC) remains the most common cause of preventable death after multiple injury. Rapid identification followed by aggressive treatment is the key for improved outcomes. Treatment of trauma hemorrhage begins at the scene, with manual compression, the use of tourniquets and (non) commercial pelvic slings, and rapid transfer to an adequate trauma center. Upon hospital admission, coagulation monitoring and support are to be initiated immediately. Bleeding is controlled surgically following damage control principles. Modern coagulation management includes goal-oriented, individualized therapies, guided by point-of-care viscoelastic assays. Idarucizumab can be used as an antidote to the thrombin inhibitor dabigatran, andexanet alpha as an antidote to factor Xa inhibitors. This review summarizes the key recommendations of the 2019 updated European guideline on the management of major bleeding and coagulopathy following trauma. These evidence-based recommendations may form the backbone of algorithms adapted to local logistics and infrastructure.

## 1. Introduction

Despite significant improvements in acute trauma care, uncontrolled hemorrhage with subsequent hemostatic derangements remain a clinical problem of highest degree [1] and the most common cause of preventable death in the acute posttraumatic phase of care [2]. Death by exsanguination occurs rapidly, at a median of 1.65 h after arrival to the hospital [3], and every fourth patient with severe trauma displays laboratory signs of coagulopathy [4]. These coagulation problems may either be present at admission or may develop in the further sequalae, thereby aggravating the overall course of the patient [5,6]. Therefore, the early identification of acute bleeds along with systemic coagulopathy followed by aggressive management are of utmost importance [7,8]. In the US PROPPR trial, for every 15 min of time reduction for bleeding/coagulopathy control, a lower rate of mortality (relative risk [RR]: 0.97; 95% confidence interval [0.94; 0.99]) and multiorgan failure (RR 0.94; [0.91; 0.97]; [9]) was observed. However, acute trauma care including measures to control and restore hemostasis is still variable even among major trauma centers [10,11,12], although the implementation and the adherence to standardized algorithms have been associated with better outcomes [13,14,15]. Meanwhile, hemostatic derangements in association with traumatic injuries are considered as an independent, multifactorial, and primary entity [5,6,10] referred to as trauma-induced coagulopathy (TIC) with a notable effect on survival [7].

Since 2005, the multidisciplinary Task Force for Advanced Bleeding Care in Trauma provides evidence-based recommendations for the management of bleeding and coagulopathy after severe injury [16]. The working Task Force consists of the core group, additional experts in hematology and guideline development, and representatives of relevant European professional societies, including the European Shock Society, the European Society for Anesthesia, the European Society for Emergency Medicine, the European Society for Intensive Care Medicine, and the European Trauma Society [16]. Recommendations (R) are based on the nominal group process and the GRADE (Grading of Recommendations Assessment, Development, and Evaluation) system of evidence [17]. Within this system, numbers stand for the level of support assigned by the expert committee and the letter for the degree of literature support for the recommendation (Table 1). By nature, the guideline issued only reflects the current knowledge and is regularly updated and revised as new evidence emerges. Since their first version published back in 2007 [16], the European guideline on the management of major bleeding and coagulopathy following trauma has been updated five times, with the latest version published in 2019 [8]. In the following, the key recommendations of the 2019 updated guidelines including changes to their previous version from 2016 are summarized.

## 2. Prehospital Management of Trauma Hemorrhage

**Figure 1:**
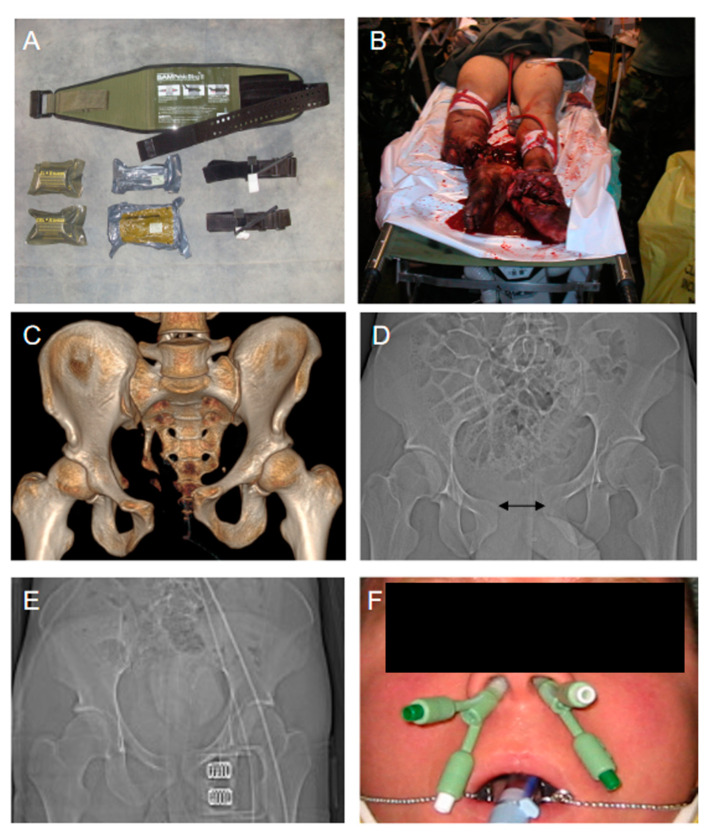
Prehospital management of traumatic bleeding. Commercial pelvic binder, tourniquets, and hemostatic dressings (**A**). Emergency bleeding control for bilateral amputation injuries via tourniquet application to both lower limbs (**B**). Pelvic closure and stabilization via use of a pelvic binder (**C**–**E**). Control of oromaxillofacial bleeding by using a blocked bladder catheter (**F**).

The treatment of acute trauma hemorrhage and co-existing hemostatic derangements, which includes TIC, begins at the scene [18]. Depending on the dynamics of blood loss, the classical ABCDE (A = Airway/Cervical Spine Protection; B = Breathing; C = Circulation; D = Disability; and E = Environment) concept is left and control of the C component (circulation/critical bleeding) gains priority even before the airway is secured according to a modified C-ABCDE concept. While there are various options for external hemorrhage control, internal hemorrhage is not seldom inaccessible for prehospital care and, in such scenarios, the immediate transfer to a designated trauma center with prompt surgical intervention remains the only valuable option [19]. Direct manual pressure applied to the tissue injury and/or supplying blood vessels are still the most effective measures for rapid bleeding control (R2/1A). In case the bleeding source is deep into the tissue the wound can be compressed and tamponed with sterile dressings. Sometimes, these manual techniques can be combined with coagulative hemostatics to locally promote blood clotting. The experience from military conflicts has shown that tourniquets can successfully control severe bleedings from injuries to the extremities (R2/1B). Their use has also been demonstrated in life-threatening hemorrhage, in case of multiple bleeding sources on one limb or in the context of concomitant and critical A, B, or C problems, if the bleeding source is inaccessible, and in mass casualty events [18]. As a matter of principle, sufficient analgesia needs to be applied in parallel and the tourniquet is attached as distal as possible, at least one hand width proximal to the injury (Figure 1). The tourniquet is to be torn and fixed until the bleeding stops and the time point of application is documented. In a retrospective study, tourniquet use was associated with improved cardiocirculatory stability at hospital admission (BPsyst 120 ± 2 vs. 112 ± 2 mm Hg, *p* = 0.003) and reduced transfusion requirement (packed red blood cells [pRBC]: 2.0 ± 0.1 vs. 9.3 ± 0.6, *p* < 0.001; fresh frozen plasma concentrates [FFP]: 1.4 ± 0.08 vs. 6.2 ± 0.4, *p* < 0.001) along with a non-increased risk for complications, e.g., nerve injuries and infections [20].

With increasing magnitude of injury, the occurrence of displaced pelvic injuries with active bleeding mostly from the venous peritoneal plexus increases; of note, only 10–15% of all pelvic bleedings are arterial [21,22]. Pelvic binders can effectively control bleeding from pelvic ring fractures (R2/1B) by reducing pelvic volume and inducing counterpressure to the bleed if applied correctly at a trochanteric level (Figure 1). Retrospective evidence has shown that initial pelvic stabilization through pelvic binders leads to fewer blood transfusions (2462 ± 2215 mL vs. 4385 ± 3326 mL; *p* < 0.01), fewer days in-hospital and on the intensive care unit (16.11 ± 12.54 vs. 19.55 ± 26.14 days and 5.33 ± 5.42 vs. 8.36 ± 11.52 days, respectively), with an overall trend towards increased survival [23]. In another retrospective study which assessed 104 patients with isolated pelvic fracture and hemodynamic instability, the mortality in the group that had received external pelvic stabilization was 19.1% versus 33.3% in the group without [24]. When considering the relatively low sensitivity and specificity for the clinical assessment of pelvic stability by hand, the decision to apply a pelvic binder should rather be liberally taken in the prehospital emergency setting. Severe oromaxillofacial bleeds can be controlled either through compression of the nostrils, packing, or, in most dramatic cases, by using a blocked bladder catheter ([18]; Figure 1). The prehospital administration of blood products remains a matter of ongoing debate; they may be context related and depend on risks and logistical challenges [25,26,27,28]. A retrospective analysis of more than 55,000 US combat datasets from the military conflicts in Iraq and Afghanistan collected between 2001 and 2017 has shown a 44% reduction in overall mortality over time, which was predominantly linked to three interventions [29]:

- Application of tourniquets.
- Limitation of prehospital transport time < 60 min.
- Early use of blood products.

## 3. Rapid Transport to Specialized Trauma Centers

The 2019 updated European guideline on the management of major bleeding and coagulopathy following trauma suggests that bleeding trauma patients should be referred directly to a designated trauma center (R1/1B). In case hemodynamic stability cannot be achieved prehospital, all further efforts on scene need to be stopped for immediate and rapid transfer of the patient to the nearest hospital [18] in order to minimize the time interval between injury and hemorrhage control (R1/1A). To prevent further blood loss, permissive hypotension is an option with systolic target pressures 80–90 mm Hg (mean target pressure 50–60 mm Hg) in the absence of traumatic brain injury (TBI) until control of bleeding has been achieved (R12/1C). In the presence of TBI, a mean arterial pressure (MAP) ≥ 80 mm Hg is suggested to maintain cerebral perfusion pressure (R12/1C). Cerebral perfusion pressure (CPP) is defined as the net pressure gradient that is necessary to drive oxygen delivery to cerebral tissues and can be calculated by the difference between MAP and intracranial pressure (ICP). Maintaining appropriate CPP in patients with intracranial pathology and deranged ICP or with hemodynamic instability may decrease the risk of further secondary ischemic brain injury. The choice of volume in hypotensive and bleeding trauma patients is still under debate but at present consists of isotonic balanced crystalloids (R15/1A); in life-threatening hemorrhage and shock, the use of vasopressors can be an option to achieve the target pressure (R14/1C).

## 4. In-Hospital Management of Traumatic Bleeding and Coagulopathy

### 4.1. Clinical Assessment and Immediate Surgical Bleeding Control

**Figure 2:**
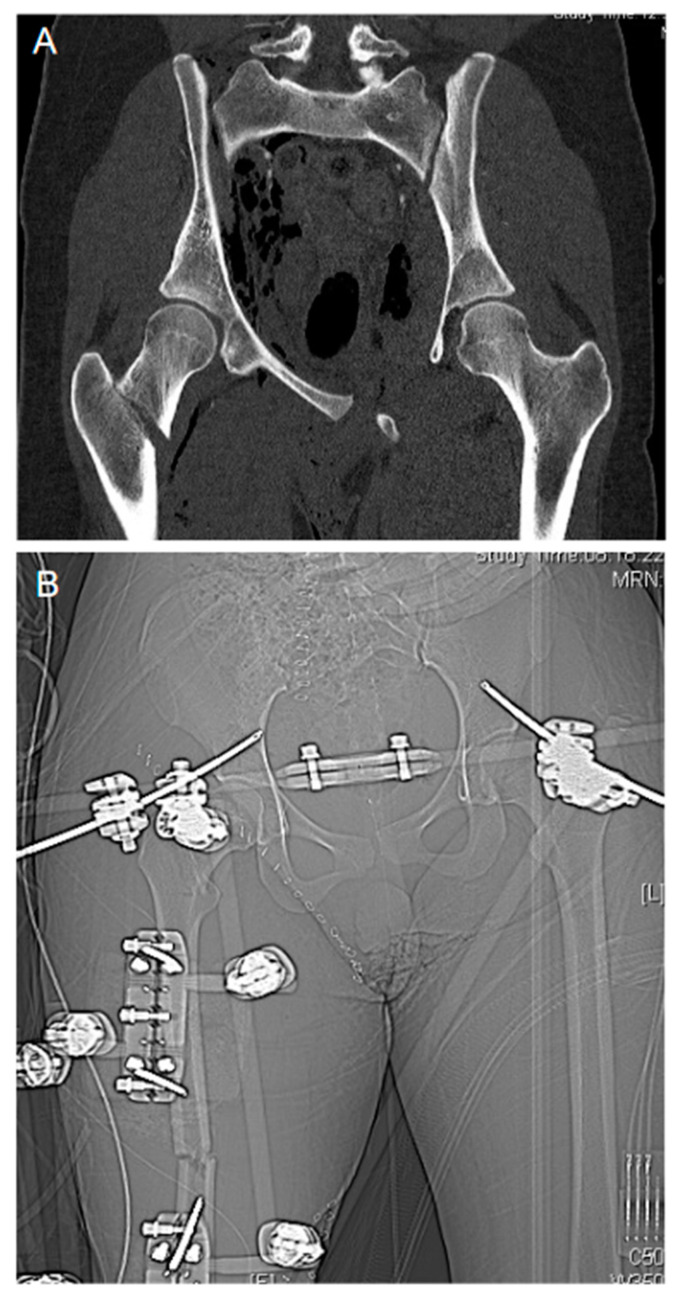
Damage control orthopedics. Emergency damage control orthopedics in a trauma case of combined displaced “open book” pelvic and right proximal femur fracture before (**A**) and after external fixation (**B**).

At hospital admission, the severity of the bleeding is estimated through a combination of the patient’s physiology, the anatomical injury and the trauma mechanism sustained (R4/1C). The Advanced Trauma Life Support (ATLS) classification of hemorrhagic shock has recently been questioned in its validity [30] and, as consequence, has been upgraded by the incorporation of the base deficit (BD) as an additional prognostic parameter in their latest version [31,32]. In the context of “permissive hypotension”, the bolus response to the administration of a defined fluid challenge is considered more and more critically (R13/1B). The role of imaging to detect free fluids in the thoracic and abdominal cavities as well as to identify and locate bleeding sources remains highlighted (FAST ultrasound [R7/1C], contrast-enhanced whole-body computed tomography [R7/1B]). Patients in which the bleeding source can be identified and those in severe hemorrhagic shock and with a suspected bleeding source are taken straight to the operation theatre for rapid bleeding control (R5/1C) according to the classical damage control procedures (R18/1B) with closure/stabilization of the pelvic ring (R19/1B) and abdominal packing (R20/1B; Figure 2). If there is an infrastructure given, angiographic embolization may be an option (R20/1B); in extremis and to gain time in life-threatening pelvic bleeding, retrograde endovascular balloon occlusion of the aorta (REBOA) may be considered until definitive care can be provided (R20/2C).

### 4.2. Rapid Detection and Diagnosis of Coagulopathies

Adequate techniques to monitor and promote coagulation should be executed immediately once the bleeding trauma patient is admitted to the trauma center (R23/1B). Low hemoglobin (Hb) in the acute phase remains an indicator for the magnitude of hemorrhage with coexisting coagulopathy (R8/1B); however, measurements are to be repeated at short intervals as an initial value within the reference ranges may mask ongoing hemorrhage (R8/1B). Indicative parameters for shock and level of hemorrhage are lactate and base deficit (BD) (R9/1A). The pragmatic work-up to screen for hemostatic derangements include the standard clotting parameters (prothrombin time [PT; Quick], platelet count, and fibrinogen concentration) and/or point-of-care PT/international normalized ratio (INR) (R10/1C) and/or functional viscoelastic testing assays (R10/1C). The 2019 updated European trauma guideline, for the first time, considers standard parameters and viscoelastic testing results as equivalent in the acute assessment of the bleeding trauma patient [8]. However, with the use of the latter, the functional clotting properties of the patient’s blood in respect to clot formation, firmness, and breakdown can be assessed as point-of-care (POC) at bedside, thereby providing a relevant time advantage [33,34]. In suspected platelet dysfunction, additional POC tests may be used to assess platelet function (R11/2C).

### 4.3. Acute “Goal-Directed” Coagulation Therapies

**Figure 3:**
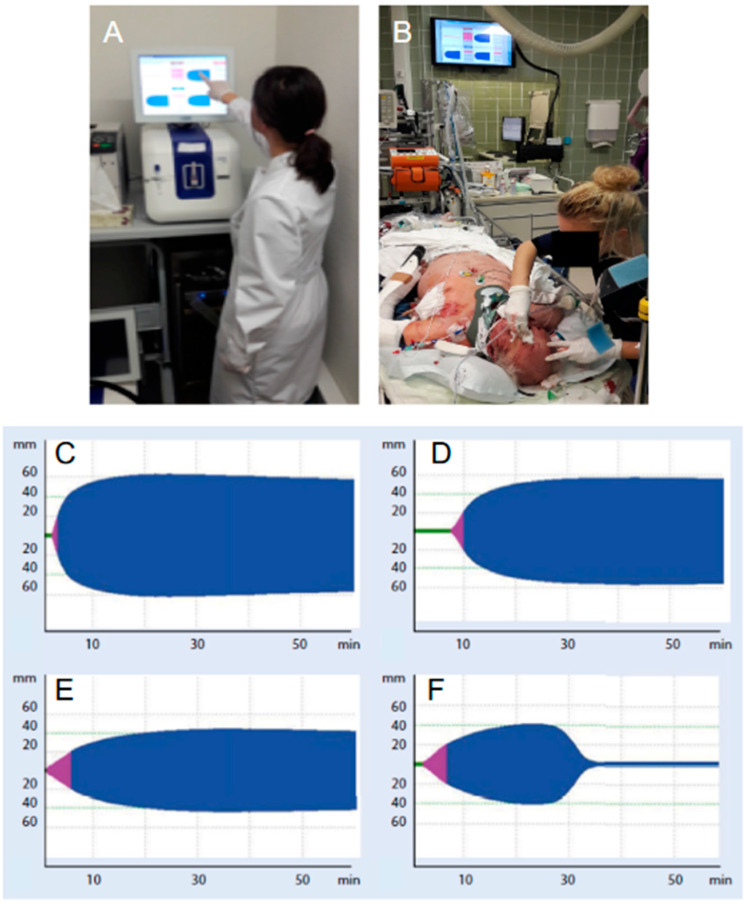
Point-of-care (POC) functional viscoelastic testing in the Emergency Department. The assay can be run at bedside or remote (**A**) and results be transferred onto a screen in the resuscitation room for rapid clinical decision making (**B**). Exemplar rotational thromboelastometric traces and interpretation. (**C**) shows a reference trace, (**D**) shows a delayed initiation of clotting with prolonged “Clotting time” (CT ↑), (**E**) shows a reduced clot firmness/stability with reduced amplitude referred to as “Maximal clot firmness” (MCF ↓), and (**F**) shows a diamond-shaped hyperfibrinolysis with clot breakdown.

**Figure 4:**
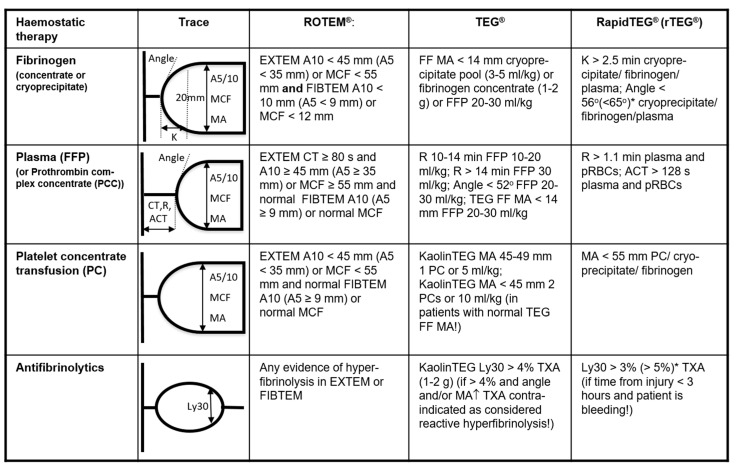
Exemplar viscoelastic-assay-driven algorithm for the use of hemostatic agents and blood products in bleeding trauma patients (modified from [36]). Overview of viscoelastic triggers for the differential and goal-directed use or lack of use of blood products and hemostatic agents based on expert opinion, for rotational thromboelastometry (ROTEM), thromboelastography (TEG), and rapid TEG (rTEG). If available, specific treatments are given (TEG and rTEG only). ROTEM parameters: EXTEM = test for the (extrinsic) hemostasis system. FIBTEM = test for the fibrin part of the clot. CT = clotting time (s). A5/A10 = clot amplitude after 5 or 10 min (mm). MCF = maximum clot firmness (mm). TEG parameters: R = reaction time (min). Angle = speed of clot formation (degrees). MA = maximum amplitude (mm). FF MA = functional fibrinogen test maximum amplitude (mm). Ly30 = amplitude reduction after 30 min as an indicator of hyperfibrinolysis (%). Additional definitions for rTEG: K = time from end of R until the clot reaches 20 mm amplitude. ACT = activated clotting time. Treatments: FFP = fresh frozen plasma. PCC = prothrombin complex concentrate. pRBCs = packed red blood cells. TXA = tranexamic acid. * Recommended trigger differ between publications.

The acute treatment where massive transfusion can be expected includes the empirical use of fresh frozen plasma (FFP) and packed red blood cell concentrates (pRBC) at a predefined ratio of at least 1:2 (R24/1C) following the damage control resuscitation (DCR) concept or, alternatively, the use of fibrinogen concentrate and pRBC (R24/1C). Both strategies should be replaced, as soon as the situation allows, by targeted and more individualized concepts, guided either by conventional standard coagulation parameters or by the results from functional viscoelastic testing assays (R25/1B). Recent studies have demonstrated a positive trend for survival and saving allogenic blood products in particular when the latter approach was followed [35]. In contrast to conventional standard coagulation tests, e.g., Quick/INR and aPTT, which only mirror the short initiation phase of the coagulation process and, therefore, only a very small proportion of generated thrombin, functional viscoelastic testing assays yield immediate information on the dynamics, the stability and the sustainability of the clot structure that is being produced including (hyper-)fibrinolysis. Functional viscoelastic testing can be performed as “point-of-care” in the resuscitation room and/or the operation theater without any delay, and the results can immediately inform therapeutic decision making ([36]; Figure 3). The Figure 4 provides a summary of consensus-based recommendations, including viscoelastic thresholds for the supplementation with hemostatic agents and blood products in trauma patients with bleeding [36]. If the treatment concept is based upon the fresh frozen plasma concentrates, their use should be guided either by the standard coagulation parameters PT and aPTT (>1.5 of normal) and/or viscoelastic patterns indicating a lack of coagulation factors (R26/1C). The administration of FFP in the absence of massive bleeding (R26/1B) or to correct hypofibrinogenemia is not advised (R26/1C). If functional viscoelastic testing is not available, the threshold for fibrinogen supplementation is ≤1.5 g/L with the Clauss method (R28/1C). The thresholds for pRBC transfusion with a hemoglobin target of 7–9 g/L (R16/1C) and for platelet concentrates with a target of >50 × 10^9^/L (R29/1C), or >100 × 10^9^/L in cases of persisting hemorrhage or traumatic injury to the brain (R29/2C), remain valid. It is self-speaking that counter-acting drops in pH and temperature be avoided (R17/1C) and that calcium levels be maintained within the reference ranges, especially in settings where a massive transfusion is needed (R30/1C). 

## 5. Hyperfibrinolysis and Tranexamic Acid (TXA)

Severe trauma almost universally triggers fibrinolysis activation and systemic hyperfibrinolysis has been identified as a key component of acute TIC with poor clinical outcomes [37]. Recent large randomized controlled trials have consistently documented that the use of the synthetic lysine analogue tranexamic acid (TXA) confers a survival advantage in a number of globally critical conditions associated with acute bleeding, including traumatic injury (CRASH-2), traumatic brain injury (CRASH-3), and post-partum hemorrhage (WOMAN), without increasing the risk for thromboembolic events [38]. According to the dose regimen in the CRASH-2 trial [39], tranexamic acid is given early to bleeding trauma patients or at risk for significant hemorrhage as 1 g bolus intravenously within 3 h of injury followed by another 1 g as infusion over 8 h (R22/1A). To date, TXA is considered a principal component to a range of massive transfusion protocols and algorithms, although the two most recent randomized trials using TXA prehospital in the setting of trauma [40] and traumatic brain injury [41] have failed to reproduce the beneficial effects of TXA seen in earlier studies for 30-day mortality and neurologic outcome at six months. However, when comparing the TXA effect stratified by time to treatment and qualifying shock severity in a post hoc comparison, 30-day mortality was lower when TXA was administered within 1 h of injury (4.6% vs. 7.6%; difference, −3.0%; 95%CI, −5.7% to −0.3%; *p* < 0.002) and in patients with severe shock (18.5% vs. 35.5%; difference, −17%; 95%CI, −25.8% to −8.1%; *p* < 0.003; [40]). While there was no increased risk of thromboembolic events observed with the conventional CRASH-2 dose regimen, these were more seen in the group that was treated with 2 g TXA bolus (9% versus 4%; [41]). In another study, a 4 g TXA bolus to severely injured patients was associated with a 32% rate of thromboembolic events [42]. The first TXA dose can be considered during transport to the receiving hospital (R22/1C). 

## 6. Fibrinogen and Coagulation Factors

Fibrinogen, also referred to as blood coagulation factor 1, is the substrate for blood to clot and the first coagulation factor which reaches critical thresholds during acute and critical bleeding. Substantial declines in fibrinogen levels have been detected in blood samples from trauma patients collected at the scene and were correlated with the magnitude of injury [43]. Fibrinogen may independently but also synergistically work with TXA in seriously injured patients who require the transfusion of blood products [44]. As a matter of principle, hyperfibrinolysis has to be inhibited before any procoagulative agents including coagulation factors are administered, e.g., fibrinogen. In a retrospective cohort analysis, TXA decreased lysis by 5.4%, while a median 3.8 g fibrinogen concentrate increased clot stability by 5.2 mm at five minutes of viscoelastic test initiation [45]. This dose corresponds to the advised initial of 3–4 g which is usually administered (R28/2C) but may be higher depending on the dynamics of hemorrhage; the subsequent doses are to be guided by viscoelastic assays and laboratory fibrinogen levels (R28/2C). The supplementation with fibrinogen concentrate or, in case fibrinogen is not locally licensed, cryoprecipitate, is advised if major bleeding coincidences with hypofibrinogenemia as detected by viscoelastic signs of a functional fibrinogen deficit or a plasma Clauss fibrinogen level ≤1.5 g/L (R28/1C). At present, the protective effects to the endothelium, e.g., glycocalyx and endothelial barrier integrity, are attributed to the fibrinogen component rather than to plasma per se [46]. The supplementation with coagulation factor XIII to promote cross-linking may be considered, but at present no clear recommendation exists (R27/2C). The administration of recombinant factor VIIa (rFVIIa) has lost attraction over the recent years and its upfront use is not advised any more (R31/1B); its use may be taken into account in ongoing hemorrhage if all other measures have failed but only if acidosis, temperature, fibrinogen level, and platelet count have been corrected beforehand (R31/2C). 

## 7. Bleeding Trauma Patients on Preinjury Anticoagulants

Demographic change is associated with a rising number of aged bleeding trauma patients on preinjury anticoagulant drugs [47]. In case preinjury treatment is secured or suspected, laboratory assessment (R10/1C) is advised, and, in case of ongoing bleeding, neutralization is recommended (R32/1C). Although the International Normalized Ratio (INR) detects the anticoagulant effect of vitamin K-dependent antagonists and neutralization can be achieved through emergency prothrombin complex concentrates (PCC) and oral vitamin K (R33/1A), the current clinical practice sees a significant trend towards the increased prescription of direct and non-vitamin K-dependent oral anticoagulants, e.g., apixaban, dabigatran, edoxaban, and rivaroxaban, for underlying cardiac and neurologic comorbidities. In the event of an acute and severe bleeding, idarucizumab is indicated as an antidote for the thrombin inhibitor dabigatran (5 g intravenously [R35/1B]), and activity levels can be monitored via thrombin time ([R35/2C] for the qualitative estimate), ecarin clotting time, and diluted thrombin time [R35/2C]) [47]. Factor-Xa inhibitors, e.g., apixaban, edoxaban, and rivaroxaban, can be assessed by chromogenic anti-factor-Xa activity tests, but these tests need to be specifically calibrated (R34/2C) [47]. In addition, these tests still remain largely unavailable, and in every hospital 24/7, they tend to react differently according to the anticoagulant in use and, even more importantly, fail to assess the complete therapeutic range. Therefore, the detection and the neutralization of the anticoagulatory effects with these novel agents remain challenging, in particular in time-sensitive emergencies [47]. In case of severe and life-threatening hemorrhage under preinjury factor-Xa inhibition, the recommended treatment is combined TXA 15 mg/kg (or 1 g) intravenously and PCC (25–50 units/kg) (R34/2C). Early in 2019, the European Medicines Agency (EMA) supported the approval of factor-Xa antidote andexanet alfa and the agent became available in most European countries. The indication for andexanet alfa is for adults on direct factor factor-Xa inhibition through apixaban or rivaroxaban when the anticoagulative effect is to be reversed quickly in scenarios of life-threatening or uncontrolled hemorrhage; of note, the agent is not licensed for prophylactic use. Andexanet alfa is used either low dose or high dose as an i.v. bolus at a target rate of 30 mg/min over 15 min or 30 min, followed by a continuous infusion of 4 mg/min or 8 mg/min for 120 min, respectively. Ongoing bleeding in patients on preinjury platelet function inhibitors or with a documented inhibition may receive platelet concentrates (R36/2C); this should be considered in particular in patients with intracranial hemorrhage in need for an acute neurosurgical intervention (R36/2B). The additional use of desmopressin under these circumstances is another option (R36/2C). Consultation with a hemostaseologist is strongly recommended.

## Figures and Tables

**Table 1:** Grading of recommendations according to [17].

Grade of Recommendation	Benefit/Risk	Evidence
1A Strong recommendation, high-quality evidence	Benefits clearly outweigh risks and vice versa	RCTs without important limitations; overwhelming evidence provided by observational studies
1B Strong recommendation, moderate-quality evidence	Benefits clearly outweigh risks and vice versa	RCTs with important limitations, e.g., inconsistent results, methodological problems, being indirect, or being imprecise; strong evidence provided by observational studies
1C Strong recommendation, (very) low-quality evidence	Benefits clearly outweigh risks and vice versa	Observational studies or case series
2A Weak recommendation, high-quality evidence	Benefits balanced with risks	RCTs without important limitations; overwhelming evidence provided by observational studies
2B Weak recommendation, moderate-quality evidence	Benefits balanced with risks and burden	RCTs with important limitations, e.g., inconsistent results, methodological problems, indirect or imprecise; strong evidence provided by observational studies
2C Weak recommendation, (very) low-quality evidence	Uncertainty for benefits and risks; both maybe closely balanced	Observational studies or case series

## Data Availability

Data sharing not applicable.
